# Supplementary material for: Variations in Growth and Photosynthetic Traits of Polyploid Poplar Hybrids and Clones in Northeast China
Source: Genes (Basel). 2022 Nov 19;13(11):2161. doi: 10.3390/genes13112161 (PMC9690688; doi:10.3390/genes13112161)
Supplement: Supplementary file 1 [file genes-13-02161-s001.zip › genes-2041688-supplementary.pdf]

Table S1 Average values of leaf traits and photosynthetic characteristics for different hybrid clones of different ploidies

| Clone/Ploidy | LL | LW | LA | LSI | Pn | Gs | Ci | Tr | WUE |
|---|---|---|---|---|---|---|---|---|---|
| BCXH | 125.87±7.31 | 139.11±4.19 | 123.4±5.08 | 0.91±0.06 | 18.91±0.79 | 0.49±0.07 | 296.44±12.86 | 5.29±0.43 | 3.60±0.39 |
| HB3.1 | 129.62±1.91 | 161.29±1.71 | 147.00±2.83 | 0.80±0.02 | 20.27±1.53 | 0.37±0.07 | 265.56±19.60 | 4.79±0.56 | 4.29±0.62 |
| HB4.1 | 117.67±1.52 | 145.71±2.30 | 124.90±2.30 | 0.81±0.01 | 19.06±1.15 | 0.30±0.05 | 253.67±12.23 | 3.81±0.48 | 5.04±0.39 |
| HY3.1 | 118.21±1.57 | 152.15±3.14 | 134.90±2.13 | 0.78±0.02 | 19.30±1.03 | 0.48±0.07 | 292.67±5.98 | 5.30±0.24 | 3.64±0.07 |
| HY3.3 | 135.71±1.83 | 179.04±2.13 | 169.60±3.21 | 0.76±0.01 | 19.09±0.83 | 0.31±0.05 | 255.78±18.25 | 4.06±0.45 | 4.76±0.54 |
| SX3.1 | 140.37±2.82 | 138.05±2.32 | 156.10±4.29 | 1.02±0.03 | 20.73±1.36 | 0.40±0.11 | 267.00±29.80 | 3.95±0.77 | 5.45±1.21 |
| SX3.2 | 130.86±3.91 | 155.99±4.01 | 160.04±2.90 | 0.84±0.01 | 19.34±1.18 | 0.55±0.07 | 301.89±3.18 | 5.34±0.40 | 3.62±0.11 |
| SX3.3 | 129.35±2.38 | 155.95±3.79 | 151.80±4.27 | 0.83±0.01 | 22.22±0.29 | 0.48±0.05 | 278.22±8.60 | 5.72±0.43 | 3.91±0.34 |
| SY3.1 | 148.44±3.10 | 160.74±2.05 | 171.60±6.19 | 0.92±0.02 | 19.81±1.84 | 0.46±0.08 | 288.11±11.81 | 3.93±0.44 | 5.08±0.56 |
| SY3.2 | 131.57±2.97 | 181.79±4.58 | 161.80±4.66 | 0.72±0.02 | 16.17±0.53 | 0.32±0.03 | 282.56±9.59 | 4.15±0.24 | 3.91±0.30 |
| XH | 131.51±2.94 | 140.34±4.64 | 127.20±6.61 | 0.94±0.02 | 19.71±1.16 | 0.49±0.03 | 292.89±5.49 | 5.39±0.20 | 3.66±0.24 |
| XH14 | 123.97±3.29 | 137.54±3.33 | 120.40±4.62 | 0.90±0.03 | 18.51±2.22 | 0.35±0.11 | 270.78±15.12 | 3.53±0.74 | 5.34±0.64 |
| XY3.1 | 108.87±3.07 | 121.41±4.27 | 91.40±5.41 | 0.90±0.05 | 18.51±2.22 | 0.35±0.11 | 270.78±15.12 | 3.53±0.74 | 5.34±0.64 |
| XY3.4 | 128.23±2.75 | 138.54±3.76 | 108.60±3.91 | 0.93±0.02 | 20.77±1.27 | 0.43±0.02 | 279.11±8.92 | 4.46±0.09 | 4.66±0.34 |
| XY3.5 | 121.43±3.04 | 129.70±3.76 | 97.20±3.96 | 0.94±0.02 | 18.93±2.15 | 0.40±0.10 | 280.44±13.21 | 4.02±0.72 | 4.78±0.57 |
| XY3.6 | 120.74±3.61 | 138.80±2.24 | 123.63±5.40 | 0.87±0.03 | 18.79±0.43 | 0.38±0.03 | 280.78±8.01 | 4.60±0.21 | 4.09±0.27 |
| XY3.7 | 117.19±3.55 | 128.89±2.45 | 104.40±6.07 | 0.91±0.02 | 18.12±0.94 | 0.29±0.06 | 255.67±17.71 | 3.43±0.54 | 5.37±0.64 |
| XY4.1 | 111.05±3.19 | 142.18±3.34 | 122.35±4.70 | 0.78±0.04 | 18.74±0.35 | 0.41±0.06 | 284.00±13.83 | 4.86±0.46 | 3.90±0.44 |
| XY4.2 | 142.45±4.48 | 161.06±6.82 | 155.20±4.76 | 0.89±0.02 | 18.59±0.33 | 0.40±0.03 | 284.89±5.30 | 5.06±0.18 | 3.68±0.13 |
| Control | 127.11±5.64 | 139.00±3.97 | 123.67±5.85 | 0.91±0.04 | 19.04±1.55 | 0.44±0.1 | 286.70±16.26 | 4.74±1.00 | 4.20±0.93 |
| Triploid | 127.74±10.52 | 149.41±18.44 | 136.77±27.96 | 0.86±0.08 | 19.39±1.91 | 0.40±0.1 | 276.81±19.16 | 4.41±0.83 | 4.53±0.83 |
| Tetraploid | 123.72±14.32 | 149.65±9.49 | 134.15±15.90 | 0.82±0.05 | 18.80±0.72 | 0.37±0.07 | 274.19±18.23 | 4.57±0.67 | 4.21±0.70 |

Note: (Pn): net photosynthesis rate; (Ci): intercellular CO2 concentration; (Gs): stomatal conductance; (Tr): transpiration rate; (WUE): water use efficiency; (LL): leaf length; (LW): leaf weight; (LA): leaf area; (LSI): leaf shape index.

Table S2 Average values of growth traits for different hybrid clones of different ploidies

| Clone/Ploidy | H1 | BD1 | H2 | BD2 | H3 | BD3 | DBH3 | V3 | H4 | BD4 | DBH4 | V4 |
|---|---|---|---|---|---|---|---|---|---|---|---|---|
| BCXH | 2.17±0.31 | 17.63±2.95 | 3.93±0.45 | 46.66±8.05 | 5.57±0.90 | 83.02±21.56 | 49.48±13.08 | 0.006±0.004 | 7.34±1.01 | 105.00±21.58 | 71.22±17.07 | 0.016±0.010 |
| HB3.1 | 1.80±0.19 | 16.45±2.28 | 2.95±0.14 | 30.88±3.97 | 4.74±0.34 | 54.25±7.25 | 33.6±4.47 | 0.002±0.001 | 7.31±0.45 | 67.22±9.48 | 44.44±4.85 | 0.006±0.002 |
| HB4.1 | 1.89±0.19 | 15.34±1.92 | 3.25±0.34 | 37.37±3.98 | 4.90±0.27 | 57.68±7.11 | 36.74±6.15 | 0.003±0.001 | 6.18±0.27 | 73.22±9.48 | 50.33±6.14 | 0.006±0.002 |
| HY3.1 | 2.07±0.17 | 16.05±1.73 | 4.00±0.26 | 42.72±4.99 | 5.70±0.38 | 77.01±8.31 | 48.21±3.70 | 0.005±0.001 | 6.69±0.33 | 98.78±9.01 | 61.22±4.71 | 0.010±0.002 |
| HY3.3 | 2.07±0.15 | 19.01±1.48 | 3.98±0.32 | 54.06±8.76 | 5.60±0.44 | 77.61±1.61 | 49.36±4.78 | 0.005±0.001 | 7.88±0.72 | 97.33±3.24 | 65.67±5.27 | 0.013±0.003 |
| SX3.1 | 2.25±0.15 | 19.75±2.31 | 4.63±0.31 | 55.53±4.45 | 6.88±0.26 | 94.54±14.59 | 64.87±7.51 | 0.011±0.003 | 9.00±0.35 | 134.11±14.04 | 94.22±8.30 | 0.030±0.006 |
| SX3.2 | 2.09±0.21 | 17.94±2.09 | 4.07±0.26 | 48.11±5.25 | 5.54±0.69 | 74.06±9.47 | 45.67±9.01 | 0.005±0.002 | 6.96±0.77 | 91.00±12.30 | 63.67±9.51 | 0.011±0.004 |
| SX3.3 | 2.07±0.13 | 18.28±1.63 | 4.22±0.26 | 50.92±2.66 | 6.20±0.63 | 82.96±11.57 | 55.16±7.96 | 0.008±0.003 | 7.77±0.87 | 101.56±12.52 | 71.33±12.85 | 0.016±0.007 |
| SY3.1 | 2.16±0.24 | 20.27±3.27 | 4.47±0.40 | 55.18±10.39 | 6.04±0.54 | 80.94±17.02 | 51.34±12.44 | 0.007±0.003 | 7.90±0.83 | 109.67±15.39 | 77.67±14.32 | 0.019±0.009 |
| SY3.2 | 2.46±0.21 | 19.61±2.26 | 4.37±0.27 | 51.10±3.99 | 5.70±0.32 | 81.78±6.33 | 49.2±7.28 | 0.005±0.002 | 6.96±0.29 | 101.89±5.25 | 66.22±9.42 | 0.012±0.004 |
| XH | 2.06±0.15 | 16.34±1.77 | 4.27±0.17 | 49.35±3.70 | 6.03±0.5 | 79.27±12.81 | 53.04±9.95 | 0.007±0.003 | 7.76±0.78 | 101.89±17.11 | 69.00±12.02 | 0.015±0.006 |
| XH14 | 2.06±0.08 | 17.00±1.26 | 4.01±0.45 | 43.95±6.86 | 6.10±0.79 | 80.92±14.48 | 52.59±9.91 | 0.007±0.003 | 7.94±0.49 | 107.11±13.31 | 69.44±10.03 | 0.015±0.005 |
| XY3.1 | 1.96±0.10 | 15.12±0.62 | 4.25±0.40 | 42.78±4.86 | 6.34±0.61 | 70.46±9.61 | 51.06±7.56 | 0.007±0.002 | 8.54±0.50 | 93.67±17.49 | 74.11±14.00 | 0.019±0.007 |
| XY3.4 | 1.75±0.08 | 14.15±1.37 | 3.29±0.28 | 33.52±2.47 | 5.58±0.08 | 59.39±1.91 | 36.43±2.26 | 0.003±0.000 | 6.98±0.43 | 78.00±8.19 | 57.89±6.53 | 0.009±0.003 |
| XY3.5 | 1.75±0.15 | 14.22±1.53 | 3.35±0.30 | 37.09±4.26 | 5.00±0.59 | 62.88±7.83 | 39.37±4.86 | 0.003±0.001 | 6.47±0.57 | 76.67±6.00 | 52.33±3.87 | 0.007±0.001 |
| XY3.6 | 1.68±0.12 | 14.48±1.21 | 3.86±0.65 | 44.09±6.14 | 5.71±0.38 | 72.45±7.63 | 43.15±4.12 | 0.004±0.001 | 7.17±0.55 | 92.22±8.00 | 64.22±8.03 | 0.012±0.004 |
| XY3.7 | 1.69±0.06 | 14.88±0.93 | 3.28±0.26 | 42.93±3.56 | 5.36±0.52 | 69.2±13.81 | 42.59±4.99 | 0.004±0.001 | 7.18±0.40 | 93.67±13.61 | 71.56±11.16 | 0.014±0.005 |
| XY4.1 | 1.74±0.18 | 15.82±1.62 | 2.73±0.25 | 30.64±3.86 | 4.14±0.78 | 49.17±16.04 | 29.45±11.25 | 0.002±0.001 | 5.78±0.83 | 79.00±12.25 | 52.11±9.74 | 0.007±0.003 |
| XY4.2 | 1.88±0.15 | 15.93±2.05 | 3.87±0.21 | 47.33±2.97 | 6.02±0.32 | 85.02±13.94 | 55.13±5.49 | 0.007±0.002 | 8.26±0.44 | 114.67±5.85 | 78.56±5.03 | 0.019±0.003 |
| Control | 2.09±0.21 | 16.99±2.12 | 4.07±0.40 | 46.65±6.67 | 5.90±0.76 | 81.07±16.14 | 51.70±10.77 | 0.007±0.003 | 7.68±0.80 | 104.67±17.11 | 69.89±12.89 | 0.015±0.007 |
| Triploid | 1.98±0.28 | 16.94±2.84 | 3.89±0.61 | 45.17±9.54 | 5.71±0.71 | 73.22±14.36 | 46.67±10.36 | 0.005±0.003 | 7.42±0.91 | 94.44±19.56 | 66.14±14.92 | 0.014±0.008 |
| Tetraploid | 1.82±0.19 | 15.66±1.81 | 3.30±0.54 | 38.85±7.52 | 5.05±0.95 | 65.26±20.11 | 41.12±13.61 | 0.004±0.003 | 6.81±1.25 | 91.44±19.52 | 61.56±14.69 | 0.011±0.007 |

Note: (H1): 1-year-old tree height; (H2): 2-year-old tree height; (H3): 3-year-old tree height; (H4): 4-year-old tree height; (BD1): 1-year-old ground diameter; (BD 2): 2-year-old ground diameter; (BD 3): 3-year-old ground diameter; (BD 4): 4-year-old ground diameter; (DBH3): 3-year-old diameter at breast height; (DBH4): 4-year-old diameter at breast height; (V3): 3-year-old volume; (V4): 4-year-old volume.

**Table S3.** Principal component analysis of three broad categories

| Principal component factor | Component I | Component II | Component III |
|---|---|---|---|
| Eigenvalue | 9.787 | 3.836 | 3.448 |
| Contribution (%) | 46.61 | 18.27 | 16.42 |
| Cumulative contribution (%) | 46.61 | 64.88 | 81.30 |
| LL | 0.468 | 0.632 | 0.071 |
| LW | -0.057 | 0.965 | 0.094 |
| LA | 0.265 | 0.920 | 0.139 |
| LSI | 0.524 | -0.575 | -0.086 |
| Pn | 0.053 | 0.014 | 0.184 |
| Gs | 0.165 | -0.012 | 0.889 |
| Ci | 0.150 | -0.035 | 0.919 |
| Tr | -0.101 | 0.183 | 0.899 |
| WUE | 0.197 | -0.240 | -0.882 |
| H1 | 0.613 | 0.547 | 0.170 |
| BD1 | 0.526 | 0.754 | 0.027 |
| H2 | 0.897 | 0.241 | 0.152 |
| BD2 | 0.833 | 0.434 | 0.047 |
| H3 | 0.936 | -0.007 | -0.016 |
| BD3 | 0.926 | 0.245 | 0.147 |
| DBH3 | 0.963 | 0.151 | 0.052 |
| V3 | 0.966 | 0.103 | 0.026 |
| H4 | 0.834 | 0.055 | -0.221 |
| BD4 | 0.940 | 0.157 | 0.058 |
| DBH4 | 0.956 | 0.003 | -0.063 |
| V4 | 0.938 | 0.003 | -0.134 |

Note: (Pn): net photosynthesis rate; (Ci): intercellular CO2 concentration; (Gs): stomatal conductance; (Tr): transpiration rate; (WUE): water use efficiency; (LL): leaf length; (LW): leaf weight; (LA): leaf area; (LSI): leaf shape index; (H1): 1-year-old tree height; (H2): 2-year-old tree height; (H3): 3-year-old tree height; (H4): 4-year-old tree height; (BD1): 1-year-old ground diameter; (BD 2): 2-year-old ground diameter; (BD 3): 3-year-old ground diameter; (BD 4): 4-year-old ground diameter; (DBH3): 3-year-old diameter at breast height; (DBH4): 4-year-old diameter at breast height; (V3): 3-year-old volume; (V4): 4-year-old volume.
